# Absences from work among healthcare workers: are they related to influenza shot adherence?

**DOI:** 10.1186/s12913-020-05585-9

**Published:** 2020-08-18

**Authors:** Francesca Antinolfi, Claudio Battistella, Laura Brunelli, Francesca Malacarne, Francesco Giuseppe Bucci, Daniele Celotto, Roberto Cocconi, Silvio Brusaferro

**Affiliations:** 1 Department of Medicine, University of Udine, Udine, Italy; 2 ULSS4 Veneto Orientale Trust, San Donà di Piave, Venezia, Italy; 3 Udine Healthcare and University Integrated Trust, Udine, Italy; 4 Friuli Centrale Healthcare University Trust, Udine, Italy; 5 Giuliano Isontina Healthcare University Trust, Trieste, Italy

**Keywords:** Influenza, Vaccination, Coverage, Healthcare workers, Absences

## Abstract

**Background:**

The coverage for influenza vaccination among healthcare workers (HCWs) is inadequate in many countries despite strong recommendations; is there evidence that influenza vaccination is effective in preventing absenteeism? Aim of the study is to evaluate the influenza vaccination coverage and its effects on absences from work among HCWs of an Italian academic healthcare trust during the 2017–2018 influenza season.

**Methods:**

We performed a retrospective study to identify predictive characteristics for vaccination, and a retrospective cohort study to establish the effect of vaccination on absences among the vaccinated and non-vaccinated cohorts between December 2017 and May 2018. Overall absence rates over the whole observation period and sub-rates over 14-days intervals were calculated; then comparison between the two groups were conducted applying Chi-square test.

**Results:**

Influenza vaccination coverage among 4419 HCWs was 14.5%. Age, university degree, medical care area and physician profile were positively associated with vaccine uptake. Globally during influenza season non-vaccinated HCWs lost 2.47/100 person-days of work compared to 1.92/100 person-days of work among vaccinated HCWs (*p* < 0.001); significant differences in absences rates resulted when focusing on the influenza epidemic peak.

**Conclusions:**

Factors predicting influenza uptake among HCWs were male sex, working within medical care area and being a physician. Absenteeism among HCWs resulted to be negatively correlated with vaccination against influenza. These findings add evidence to the urgent need to implement better influenza vaccination strategies towards HCWs to tackle vaccine hesitancy among professionals.

## Background

Influenza is an acute and highly contagious illness caused by type A and B influenza viruses, which leads every year in epidemics with increasing morbidity and mortality, particularly among high-risk groups [1]. The World Health Organization (WHO) estimates that annual epidemics are responsible for 3–5 million cases of severe illness and between approximately 290,000 and 650,000 respiratory disease-related deaths worldwide [2]. Annual vaccination represents the most effective way to prevent influenza and is thus strongly recommended to those at high risk of complications, as well as to people who live with or care for people at high risk [1].

Studies estimate that during a mild influenza season 23% of healthcare workers (HCWs) get infected, although only 41–72% of them showing clinical symptoms; the remaining develop a subclinical, though still potentially transmissible, form of influenza [3]. The vaccination against influenza of this group of workers, along with protecting the single HCW from acquiring the disease, also enhance safety of patients who are at greater risk of severe complications, such as the elderly, individuals with diabetes or other chronic conditions [4–7]. In fact, the diffusion of influenza among HCWs could anticipate the spread of the disease among general population [8]. Once infected, an HCW may spread the virus to patients and colleagues resulting in an hospital outbreak, but to the community as well, finally increasing hospitalizations or even deaths, and therefore contributing to increase costs for the healthcare system [4, 5, 9]. For these reasons WHO, CDC (Centers for Disease Control and Prevention) and ECDC (European Centre For Disease Control and Prevention) strongly recommend annual vaccination against influenza for all HCWs [1, 10, 11]. In Italy, the 2017–2019 National Vaccination Prevention Plan (PNPV) has identified HCWs as well as a target category for influenza vaccination as well, confirming its multiple aims toward patient and community’s protection, but also in assuring the good functioning of health services during influenza season [12]. Despite strong recommendations to raise coverage among HCWs [1], compliance with influenza vaccination is still inadequate in many European countries [13] including Italy [13–17].

Another aspect to be considered is the possible economic impact of the HCWs’ absence from work due to influenza, with possible healthcare services disruption and costs increase [18]. The point is: is there evidence that influenza vaccination is effective in preventing absenteeism due to illness?

The relationship between the two has been investigated by some Authors, but with no clear agreement. On one hand, Wilde et al. found that influenza vaccination did not reduce absenteeism among HCWs [19] and the study of Gianino et al. seems to confirm this evidence in the Italian context as well [20]. On the other hand, non-specific [21] and ILI-related [22] absenteeism among HCWs were significantly reduced by influenza vaccination; this evidence was confirmed by the recent Costantino et al. work which found a correlation between the increase in influenza vaccination coverage and the reduction of lost days of work [23].

The primary aim of this study is to evaluate the influenza vaccination coverage among HCWs of an Italian academic healthcare Trust during the 2017–2018 influenza season; the secondary aim is to identify predictive characteristics of vaccination adherence; finally, the third aim is to evaluate the effects of influenza vaccination on HCWs’ absences from work.

## Methods

During 2017, from November 6th to December 6th, the vaccination campaign against influenza was conducted within the Udine Healthcare and University Integrated Trust (Italy) throughout administration to HCWs of the quadrivalent split-virion influenza vaccine (Vaxigrip Tetra).

### Data collection

Data about sex, age, educational level, professional profile, department, unit and (when the case) end of employment of HCWs working within Udine Healthcare and University Integrated Trust were collected. Using employee’s anonymous identification number, data were linked to the 2017 influenza vaccination records. Two groups were identified: vaccinated and non-vaccinated HCWs. Professional profiles were coded as: physicians, nurses, midwifes, healthcare collaborators (including public health nurses, dietitians, physiotherapists, pediatric nurses, speech therapists, optometrists and psychologists) and auxiliary personnel. Departments and units were grouped by care area in medical, surgical, intensive, healthcare services, primary & community, organization & governance, and mixed. Medical residents, medical students, trainees and office workers were not included in the analyses. HCWs’ data were merged with a database reporting HCWs’ absences from work for any reason occurred between December 11th, 2017 (the first Monday following the start of the vaccination offer) and May 13th, 2018 (the last Sunday, 1 month after the end of the influenza season).

### Statistical analysis

Categorical variables were reported as frequencies and percentages. Continuous variables were reported as mean ± standard deviation (SD) or median with inter-quartile range, depending on data distribution. The Shapiro-Wilk test was used to study data distribution. For categorical variables, Chi-square or Fisher exact tests were conducted to detect significant differences between vaccinated and non-vaccinated group, as appropriate. The Student’s t or the Mann-Whitney U tests were used to compare continuous variables between the two groups, as appropriate. Univariate and multivariate logistic regression were performed to identify the predictive variables for vaccination. All variables with *P*-value < 0.05 were included in the multivariate regression model, according with backward stepwise selection method. To compare absences between vaccinated and non-vaccinated HCWs, absence rates were calculated by dividing the number of person-days lost due to absence, over the number of scheduled working person-days within the two groups. HCWs who have seen their employment contract terminated on a given date were dropped out from observation and were excluded from the denominator starting from that date. Overall rates were calculated over the whole observation period (from December 11th, 2017 to May 13th, 2018) and then sub-rates were calculated over 14-days time intervals. The comparisons between absence rates of the two groups were performed applying Chi-square test. The Bonferroni correction was applied when considering the comparisons over the 14-days time intervals (11 intervals). The adjusted type I error (α_a_) was fixed dividing the original type I error (α_o_ = 0.05) by 11 = 0.005. The 95% (or 99.5% when Bonferroni was applied) confidence intervals for absence rates were calculated using the Wilson score method without continuity correction [24]. The confidence intervals for the differences between two rates were calculated applying the Newcombe-Wilson method without continuity correction [25]. Statistical significance for all the other tests was set accepting a type I error α < 0.05. All statistical analyses were performed using Stata/IC 13.0 (StataCorp LP, College Station, USA). The study was conducted in accordance with all national regulations, with the principles of the Declaration of Helsinki and it was approved by the Institutional Review Board of the University of Udine.

## Results

### Influenza vaccination coverage

According to administrative records, during the 2017 influenza vaccination campaign a total of 4419 HCWs were employed at the Udine Healthcare and University Integrated Trust. The characteristics of the employees are summarized in Table 1. During the vaccination campaign, 641 of them (14.5%) were vaccinated for influenza. HCWs receiving the influenza shot were older (median age 50 years [range Q_1_ – Q_3_: 43–57]) than the others, whose median age was 46 years ([range Q_1_ – Q_3_: 38–53], *p* < 0.001). Vaccination compliance was higher for males (20.7% vs 12.6%; p < 0.001), and for those having a university degree (17.0%; p < 0.001), compared with other employees having high school (13.2%), primary or lower secondary education (12.2%). Vaccination coverage rate resulted to be different also comparing professional profiles and care areas, as summarized in Table 1.

**Table 1:** Characteristics of the population of HCWs

Characteristics		Vaccinated HCWs n. (column %)	Total HCWs n. (row %)
Care area	Healthcare services	62 (25.3)	245 (5.5)
	Intensive	51 (14.4)	355 (8.0)
	Medical	237 (16.7)	1418 (32.1)
	Organization & governance	101 (11.5)	875 (19.8)
	Primary & community	85 (13.8)	615 (13.9)
	Surgical	87 (11.3)	771 (17.5)
	Mixed	18 (12.9)	140 (3.2)
Educational qualification	Primary or lower secondary education	140 (12.2)	1143 (25.9)
	High school / Upper secondary education	194 (13.2)	1466 (33.2)
	Bachelor’s or equivalent level	307 (17.0)	1810 (41.0)
Employment	Permanent	610 (14.5)	4205 (95.2)
	Temporary	31 (14.5)	214 (4.8)
Healthcare service	Extra-hospital	139 (13.9)	999 (22.6)
	Intra-hospital	502 (14.7)	3420 (77.4)
Professional profile	Auxiliary personnel	81 (9.2)	878 (19.9)
	Healthcare collaborators	95 (13.8)	687 (15.6)
	Midwives	2 (3.9)	51 (1.2)
	Nurses	240 (11.9)	2018 (45.7)
	Physicians	223 (28.4)	785 (17.8)
Sex	Female	427 (12.6)	3386 (76.6)
	Male	214 (20.7)	1033 (23.4)

### Protective factors for vaccine uptake

At the univariate logistic regression analysis, age and university degree were positively associated with influenza vaccination (respectively Crude OR = 1.037 [95%CI: 1.028–1.046], *p* < 0.001 and Crude OR = 1.463 [95%CI: 1.180–1.815], *p* = 0.001), while female sex resulted to be negatively associated (Crude OR = 0.552 [95%CI: 0.461–0.662], p < 0.001). There was no association with temporary/permanent nature of the employment (Crude OR = 0.998 [95%CI: 0.676–1.475], *p* = 0.993), nor with intra/extra-hospital healthcare service (Crude OR = 1.064 [95%CI: 0.869–1.304], *p* = 0.546). At the same time, nurses (Crude OR = 0.340 [95%CI: 0.277–0.418], *p* < 0.001), healthcare collaborators (Crude OR = 0.404 [95%CI: 0.310–0.528], p < 0.001), auxiliary personnel (Crude OR = 0.256 [95%CI: 0.194–0.338], p < 0.001), and midwives (Crude OR = 0.103 [95%CI: 0.025–0.427], *p* = 0.002) were less likely to be compliant to influenza vaccination than physician. When compared to medical care area, both surgical and organization & governance care areas were negatively related to vaccination, respectively with Crude OR = 0.634 ([95%CI: 0.487–0.825], *p* = 0.001) and Crude OR = 0.650 ([95%CI: 0.506–0.835], p = 0.001). On the contrary, healthcare services area was positively related to vaccination compliance when compared to medical area (Crude OR = 1.688 [95%CI: 1.226–2.325], p = 0.001). No differences were found for intensive care (Crude OR = 0.836 [95%CI: 0.602–1.160], *p* = 0.284), primary & community care (Crude OR = 0.799 [95%CI: 0.611–1.045], *p* = 0.101), nor mixed care (Crude OR = 0.735 [95%CI: 0.440–1.229], *p* = 0.241) when compared to medical care area.

For the multivariate logistic regression analysis, all variables that were found to have a significant association with influenza shot at univariate analysis were included (sex, age, educational qualification, professional profile and care area). A negative association with influenza vaccination was confirmed for the following: female vs male sex, surgical vs medical area, all professional profiles (nurses, healthcare collaborators, auxiliary personnel and midwives) vs physicians. Crude and adjusted odds ratios and confidence intervals for each category are reported in Table 2. A positive association with influenza vaccination in the model was found for age, considered as a continuous variable (Adjusted OR = 1.032 [95%CI: 1.023–1.042], *p* < 0.001).

**Table 2:** Univariate and multivariate logistic regression model considering vaccination as dependent variable

Characteristics		Crude OR (95%CI)	***p***-value	Adjusted OR^a^ (95%CI)	***p***-value
Age (modeled as continuous)		1.037 (1.028–1.046)	< 0.001	1.032 (1.023–1.042)	< 0.001
Care area	Medical	1	–	1	–
	Surgical	0.634 (0.487–0.825)	0.001	0.662 (0.514–0.853)	0.001
	Intensive	0.836 (0.602–1.160)	0.284		
	Healthcare services	1.688 (1.226–2.325)	0.001		
	Primary & community	0.799 (0.611–1.045)	0.101		
	Organization & governance	0.650 (0.506–0.835)	0.001		
	Mixed	0.735 (0.440–1.229)	0.241		
Educational qualification	Primary or lower secondary education	1	–		
	High school / Upper secondary education	1.093 (0.866–1.379)	0.455		
	Bachelor’s or equivalent level	1.463 (1.180–1.815)	0.001		
Employment	Permanent	1	–		
	Temporary	0.998 (0.676–1.475)	0.993		
Healthcare service	Extra-hospital	1	–		
	Intra-hospital	1.064 (0.869–1.304)	0.546		
Professional profile	Physicians	1	–	1	–
	Healthcare collaborators	0.404 (0.310–0.528)	< 0.001	0.424 (0.321–0.560)	< 0.001
	Nurses	0.340 (0.277–0.418)	< 0.001	0.413 (0.329–0.518)	< 0.001
	Auxiliary personnel	0.256 (0.194–0.338)	< 0.001	0.267 (0.199–0.357)	< 0.001
	Midwives	0.103 (0.025–0.427)	0.002	0.168 (0.040–0.705)	0.015
Sex	Male	1	–	1	–
	Female	0.552 (0.461–0.662)	< 0.001	0.809 (0.660–0.993)	0.043

^a^The non-associated variables were automatically excluded from the multivariate model by backward-stepwise selection method. All crude and adjusted ORs are shown

### Absenteeism during influenza season

**Fig. 1:**
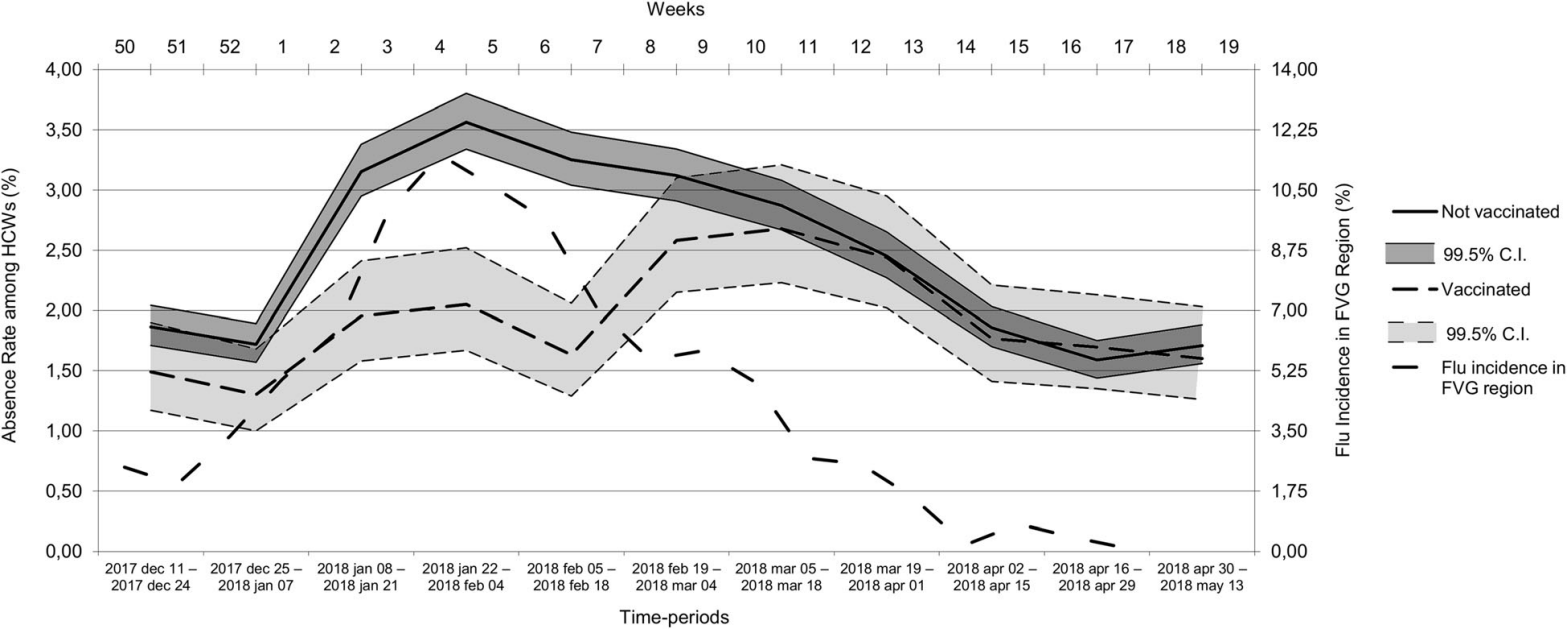
Absence rate among vaccinated and not vaccinated HCWs over time, divided in 14 days-long periods. The graph shows the 99.5% confidence intervals for each rate

At the first day of data collection about absences from work, there were a total of 4382 HCWs, as 37 employees of the initial population had dropped out from the cohort due to turn-over, new employment, retirement or other causes. At the end of the observation period there were 4280 HCWs, 620 of them having been vaccinated; the drop-out rate was not different between vaccinated and non-vaccinated groups. Globally, from December 11th, 2017 to May 13th, 2018, non-vaccinated HCWs lost 2.47 person-days / 100 person-days of work, compared to 1.92 person-days / 100 person-days of work among vaccinated HCWs (*p* < 0.001). Complete data are summarized in Table 3. Considering 14 days-long intervals, the largest differences between non-vaccinated and vaccinated HCWs were found between January 08th and 21st (Δ = + 1.20%, 99.5%CI: [0.70% – 1.64%], *p* < 0.001), January 22nd and February 04th (Δ = + 1.52%, 99.5%CI: [1.00% – 1.97%], p < 0.001), and February 05th and 18th (Δ = + 1.62%, 99.5%CI: [1.15% – 2.03%], p < 0.001). A visual representation of the absence rates of the two groups over time is shown in Fig. 1.

**Table 3:** Comparison of the absence rate between vaccinated and not vaccinated HCWs in the considered period

Year	Period	Non-vaccinated		Vaccinated		Δ Absence rate %	Confidence intervals^a^	***p***-value^**†**^
		Absence person-days/Total person-days	Rate%	Absence person-days/Total person-days	Rate%			
2017	dec 11 – dec 24	978/52,477	1.86	132/8862	1.49	+ 0.37	−0.06 – 0.74	0.015
2017–2018	dec 25 – jan 07	899/52,308	1.72	115/8842	1.30	+ 0.42	0.0001–0.76	**0.004**
2018	jan 08 – jan 21	1644/52,139	3.15	172/8812	1.95	+ 1.20	0.70–1.64	**< 0.001**
	jan 22 – feb 04	1855/52,040	3.56	180/8784	2.05	+ 1.52	1.00–1.97	**< 0.001**
	feb 05 – feb 18	1689/51,928	3.25	143/8764	1.63	+ 1.62	1.15–2.03	**< 0.001**
	feb 19 – mar 04	1617/51,811	3.12	226/8756	2.58	+ 0.54	−0.02 – 1.03	0.007
	mar 05 – mar 18	1484/51,687	2.87	234/8736	2.68	+ 0.19	−0.37 – 0.69	0.317
	mar 19 – apr 01	1265/51,619	2.45	213/8735	2.44	+ 0.01	−0.53 – 0.48	0.944
	apr 02 – apr 15	956/51,510	1.86	154/8722	1.77	+ 0.09	−0.38 – 0.49	0.562
	apr 16 – apr 29	817/51,437	1.59	148/8722	1.70	−0.11	−0.57 – 0.27	0.456
	apr 30 – may 13	876/51,269	1.71	139/8683	1.60	+ 0.11	−0.34 – 0.48	0.471
Overall		14,080/570,225	2.47	1856/96,418	1.92	+ 0.54	0.45–0.64	**< 0.001**

^a^ The 99.5% C.I. was adopted when comparing absence rates along the 14 days-long periods to comply with the Bonferroni correction. The 95% C.I. was adopted when comparing absence rates of the overall period

† The differences in absence rates are considered statistically significant when *p* < 0.005 according to the Bonferroni correction, except in the overall period comparison

## Discussion

During the 2017–18 vaccination campaign, the proportion of our HCWs getting their influenza shot was quite limited (14.5%), despite being similar to what reported for the Italian [14, 26–30] and international context [31]. Low vaccine coverage among HCWs is well known and confidence, complacency and convenience factors have been reported to contribute to vaccine hesitancy [32]. In our case, uptake among nurses, auxiliary personnel and midwives are particularly alarming, considering on one hand the direct and prolonged contact these professionals have with high-risk patients, and on the other hand their missing role as vaccine promoters [33–35].

Professional profile is confirmed to be an important influenza vaccination predictor among HCWs [14, 15, 17, 27], as well as age [15, 17, 28] and male [17, 28]. Results seem to be still uncertain also concerning intensive care area as Esposito et al. [17] found HCWs working in emergency units to be more likely to undergo influenza vaccination when compared to HCWs working in medical department, but different aggregation of workers were made in the two studies making result not comparable. The detailed analysis on HCWs vaccination adherence allowed the hospital leadership to improve the influenza vaccination strategy, by focusing on less adherent HCWs categories: during 2018–19, targeted education was given to nurses and midwifes, and on-site vaccination days were set to tackle convenience-related issues of specific care areas.

During the observation period, absenteeism work in vaccinated HCWs resulted to be far lower than among non-vaccinated colleagues, either considering the whole observation period or focusing on the 2017–18 influenza epidemic peak [36], thus confirming the existing relation between HCWs influenza vaccination coverage and absenteeism [19, 21–23, 37–39]. Even if some colleagues suggest the existence of a ceiling effect when vaccine coverage is over 40% [40], Italian and European results are still under this risk rate and therefore more efforts are needed to improve adherence. Such a detailed analysis of vaccination adherence should be performed each year by healthcare institutions to identify specific existing gaps in term of both magnitude and features to be tackled in the following influenza season. Moreover, the confirmation of both efficacy and effectiveness of influenza vaccination based on local data, could be important elements to include when presenting the influenza vaccination campaign to healthcare professionals to tackle vaccine hesitancy, as already suggested by Pereira et al. [38].

Vaccination against influenza can play a fundamental role in pursuing the reliability of healthcare services during influenza season [41] and is therefore an essential goal for healthcare organizations. As long as influenza vaccination is not mandatory for HCWs in Italy, the combination of factors predicting influenza uptake, vaccine hesitancy determinants and the most suitable interventions to put in place [42] should all be considered while planning strategies for the annual campaign against influenza within healthcare institutions.

This study has some limitations: firstly, the retrospective study design has intrinsic limitations. Secondly, we considered absences from work for any reason, without being able to distinguish clinical from other reasons underlying the absenteeism. Even if the provision of a medical certificate justifying absence is mandatory for employees, according to Italian law the employer cannot access those clinical contents. Nevertheless, we have no reason to believe that the distribution of absence causes was different between the two groups, therefore equally distributing this potential bias. Thirdly, we could not control for potential confounders about HCWs’ health status or risky behaviors (e.g. smoking habits). Finally, the results presented in this study are based on data collected in 2018 for improvement purposes on HCWs vaccination campaign within the hospital. Since 2018 late summer when these analyses were presented to the leadership, several changes have been made to foster personnel compliance to the subsequent influenza vaccination campaigns held in 2019 and 2020. Despite these limitations, our study has strength points including the choice of observing all HCWs of our trust throughout the entire 2017–18 influenza season. In addition, several variables were considered for the multivariate analysis to detect predictive HCWs characteristics for the influenza uptake and the great detail of uptake time trend allowed us to accurately analyze the relationship between vaccination against influenza and absenteeism.

## Conclusions

Our results showed that factors predicting influenza uptake among healthcare workers were male sex, working within medical care area and being a physician. Absenteeism among healthcare workers resulted to be negatively correlated with vaccination against influenza. These findings add evidence to the urgent need to implement better influenza vaccination strategies towards healthcare workers to tackle vaccine hesitancy among professionals, as this target population can at the same time protect patients, preventing them to acquire influenza and also being a positive example for the community.

## Data Availability

The datasets used and/or analysed during the current study are available from the corresponding author on reasonable request.

## Abbreviations

HCWs: Healthcare workers
WHO: World Health Organization
CDC: Centers for Disease Control and Prevention
ECDC: European Centre for Disease Control and Prevention
PNPV: National Vaccination Prevention Plan
ILI: Influenza-like illness
SD: Standard deviation
